# Osthole Stimulated Neural Stem Cells Differentiation into Neurons in an Alzheimer's Disease Cell Model via Upregulation of MicroRNA-9 and Rescued the Functional Impairment of Hippocampal Neurons in APP/PS1 Transgenic Mice

**DOI:** 10.3389/fnins.2017.00340

**Published:** 2017-06-13

**Authors:** Shao-Heng Li, Peng Gao, Li-Tong Wang, Yu-Hui Yan, Yang Xia, Jie Song, Hong-Yan Li, Jing-Xian Yang

**Affiliations:** ^1^Department of Pharmacology, School of Pharmacy, Liaoning University of Traditional Chinese MedicineDalian, China; ^2^Department of Anesthesiology, First Affiliated Hospital of Dalian Medical UniversityDalian, China; ^3^Department of Neurological Rehabilitation, Second Affiliated Hospital of Dalian Medical UniversityDalian, China; ^4^Department of Engineering, University of OxfordOxford, United Kingdom

**Keywords:** osthole, Alzheimer's disease, amyloid precursor protein, neural stem cell, microRNA-9

## Abstract

Alzheimer's disease (AD) is the most serious neurodegenerative disease worldwide and is characterized by progressive cognitive impairment and multiple neurological changes, including neuronal loss in the brain. However, there are no available drugs to delay or cure this disease. Consequently, neuronal replacement therapy may be a strategy to treat AD. Osthole (Ost), a natural coumarin derivative, crosses the blood-brain barrier and exerts strong neuroprotective effects against AD *in vitro* and *in vivo*. Recently, microRNAs (miRNAs) have demonstrated a crucial role in pathological processes of AD, implying that targeting miRNAs could be a therapeutic approach to AD. In the present study, we investigated whether Ost could enhance cell viability and prevent cell death in amyloid precursor protein (APP)-expressing neural stem cells (NSCs) as well as promote APP-expressing NSCs differentiation into more neurons by upregulating microRNA (miR)-9 and inhibiting the Notch signaling pathway *in vitro*. In addition, Ost treatment in APP/PS1 double transgenic (Tg) mice markedly restored cognitive functions, reduced Aβ plague production and rescued functional impairment of hippocampal neurons. The results of the present study provides evidence of the neurogenesis effects and neurobiological mechanisms of Ost against AD, suggesting that Ost is a promising drug for treatment of AD or other neurodegenerative diseases.

## Introduction

Alzheimer's disease is the most common degenerative disease that occurs during presenium and senectitide and is characterized by progressive dementia (Park et al., 2012; Gu et al., 2015). The major pathological hallmark of AD is a serious loss of neurons and synapses in the brain (Selkoe, 2002; Haass and Selkoe, 2007). Current treatments for AD primarily focus on the use of actylcholinesterase inhibitors designed to inhibit the enzyme acetyl cholinesterase to elevate Ach levels (Zhang et al., 2010). However, these drugs are only able relieve AD symptoms and have deleterious side effects that limit success (Iqbal and Grundke-Iqbal, 2010). Therefore, there is an urgent need for an effective therapeutic agent for AD.

Ost (7-methoxy-8-isopentenoxycoumarin, C_15_H_16_O_3_, 244.39 Da), which is a natural coumarin derivative isolated from *Cnidium monnieri* (L.) Cusson, has attracted much attention because of its potent neuroprotective activities via anti-oxidant, anti-inflammatory, and anti-apoptotic effects *in vitro* and *in vivo* (Chen et al., 2011; Gao et al., 2014; Kong et al., 2015; Xia et al., 2015). Previous studies have shown that Ost protects against neuronal death in APP-expressing NSCs (Yao et al., 2015), providing a potent agent for the treatment of AD.

NSCs are a specific type of multipotent stem cells that are present in the hippocampus and subventricular zone (SVZ) of the brain (Bacigaluppi et al., 2009). The capability of neuronal differentiation makes neurons attractive candidates for cell replacement therapy, not only in AD, but also in other neurodegenerative diseases (Yang et al., 2009). A host of studies has indicated that transplantation NSCs differentiate into neurons and astrocytes as well as improve the learning and memory deficits in AD models (Park et al., 2012; Zhang et al., 2014). However, the mechanism of NSCs differentiation into neurons remains undetermined in AD.

miRNAs are a recently identified large family of 21–23 nucleotide non-coding RNAs that are involved in numerous cellular processes, including development, proliferation, differentiation and apoptosis (Lagos-Quintana et al., 2002). Quiet a few miRNAs, including miR-9, are specifically expressed in the neurogenic regions of the brain during neural development and in adulthood (Coolen et al., 2013; Meza-Sosa et al., 2014). Recent studies have investigated the reduction of miR-9 in AD models (Schonrock et al., 2010a; Che et al., 2014). In a previous study, we demonstrated that Ost exert a functional protective role in the neuronal synapse through upregulation of miR-9 in APP-overexpressing neural cells (Li et al., 2016). However, whether Ost is able to promote APP-overexpressing NSCs to differentiate into neurons remains unclear.

The Notch pathway is an intercellular signaling mechanism that regulats a variety of biological characteristics of NSCs, including cell self-renewal, cellular differentiation and death (Imayoshi et al., 2010). Recent studies suggested that the Notch pathway, particularly Notch 1 signaling, influences the disease process in AD (Lathia et al., 2008). The fact that the presenilin-γ-secretase complex can cleave APP to generate Aβ and also cleave Notch 1 to generate Notch intracellular domain (NICD) has elucidated the role of Notch 1 in AD (Okochi et al., 2006). Moreover, APP induces glial differentiation of NPCs through Notch signaling and the basic helix-loop-helix transcription factor Hes 1(Kwak et al., 2011), the target site of miR-9 (Jing et al., 2011). Consequently, we hypothesized that Ost might improve the differentiation efficiency of APP-expressing NSCs by upregulating miR-9 and inhibiting the Notch signaling pathway.

In the present study, we established a cell model through stable transduction of APP in NSCs, which can mimic the characteristics of AD. Ost stimulated APP-expressing NSCs to differentiate into more neurons by upregulating miR-9 and inhibiting the Notch signaling pathway. In addition, we showed the capacity of Ost to improve cognitive function and rescue the functional impairment of hippocampal neurons in APP/PS1 transgenic mice.

## Materials and methods

### Generation of NSCs

NSCs were isolated from the SVZ region of newborn (0–2 days) C57BL/6 mice and cultured in NSCs proliferation media as previously describe in our laboratory (Yang et al., 2009, 2012). Briefly, the SVZ regions of the fresh brains were isolated and cut into 1-mm^3^ pieces and subsequently suspended in 3 mL of 0.25% trypsin-EDTA (Invitrogen) at 37°C for 15 min. After filtration through a 70-μm cell strainer (BD Falcon), the cells (1 × 10^6^/ml) were plated onto poly-L-lysine coated 24-well plates (BD Bioscience, San Jose, CA) and maintained in a humidified atmosphere (5% CO_2_-95% air) at 37°C. NSCs were cultured in Dulbecco's modified Eagle's medium (DMEM)/F12 (Gibco, Grand Island, USA) supplemented with 2% B27 (Gibco), 20 ng/mL epidermal growth factor (EGF, Peprotech, Rocky Hill, NJ, USA), and 20 ng/mL basic fibroblast growth factor (b-FGF, Peprotech) with 100 IU/mL penicillin and 100 μg/mL Streptomycin (Sigma) in a humidified atmosphere at 37°C. Neurospheres were formed after 3–5 days of culture. For passaging, free-floating neurospheres were collected and mechanically dissociated into small neurospheres or single cells and reseeded at a density of 10^6^ cells/mL in NSCs proliferation medium. NSCs at passage 3–8 were used in the following experiments.

### Construction of lentiviral vector encoding APP, GFP, and transduction into NSCs

To generate APP expression construct, we subcloned the human APP_695swe_ (APP) sequence (AuGCT DNA-SYN Biotechnology Co. Ltd. Beijing, China) into the GFP lentiviral vector pCDH-CMV-MCS-EF1-copGFP (System Biosciences; Mountain View, CA) at XbaI and NotI restriction sites (Invitrogen). The newly generated APP and three other helper plasmids pLP1, pLP2, and pLP/VSV-G (Invitrogen) were isolated from bacteria with the plasmid small kit without endotoxin (Omega Bio-tek; Norcross, GA), and their concentration was adjusted to 1 μg/μl. The 293T cells (Dalian Medical Unversity, Dalian, China) were cultured in DMEM supplemented with 10% FBS and 1%P/S. Ninety percent of confluent 293T cells in DMEM were transfected with plasmid DNA containing 15 μg APP or 15 μg GFP (negative control vector), 6.5 μg pLP1, 2.5 μg pLP2, and 3.5 μg pLP/VSV-G with Lipofectamine 2000 (Life Technologies, USA) in 10-cm dishes. After 6 h, the medium with plasmids was replaced by 10 ml fresh culture medium. The culture media were harvested after 2 days and filtered through a 0.45 μm membrane, then stored at −80°C for further use (Yao et al., 2015; Jiao et al., 2016). Lentiviral particles encoding APP-GFP and GFP were respectively transduced into NSCs. After 3 days, the stably transduced cells were determined by immunocytochemistry staining or cultured for future use.

### Preparation of Ost

**Figure 1:**
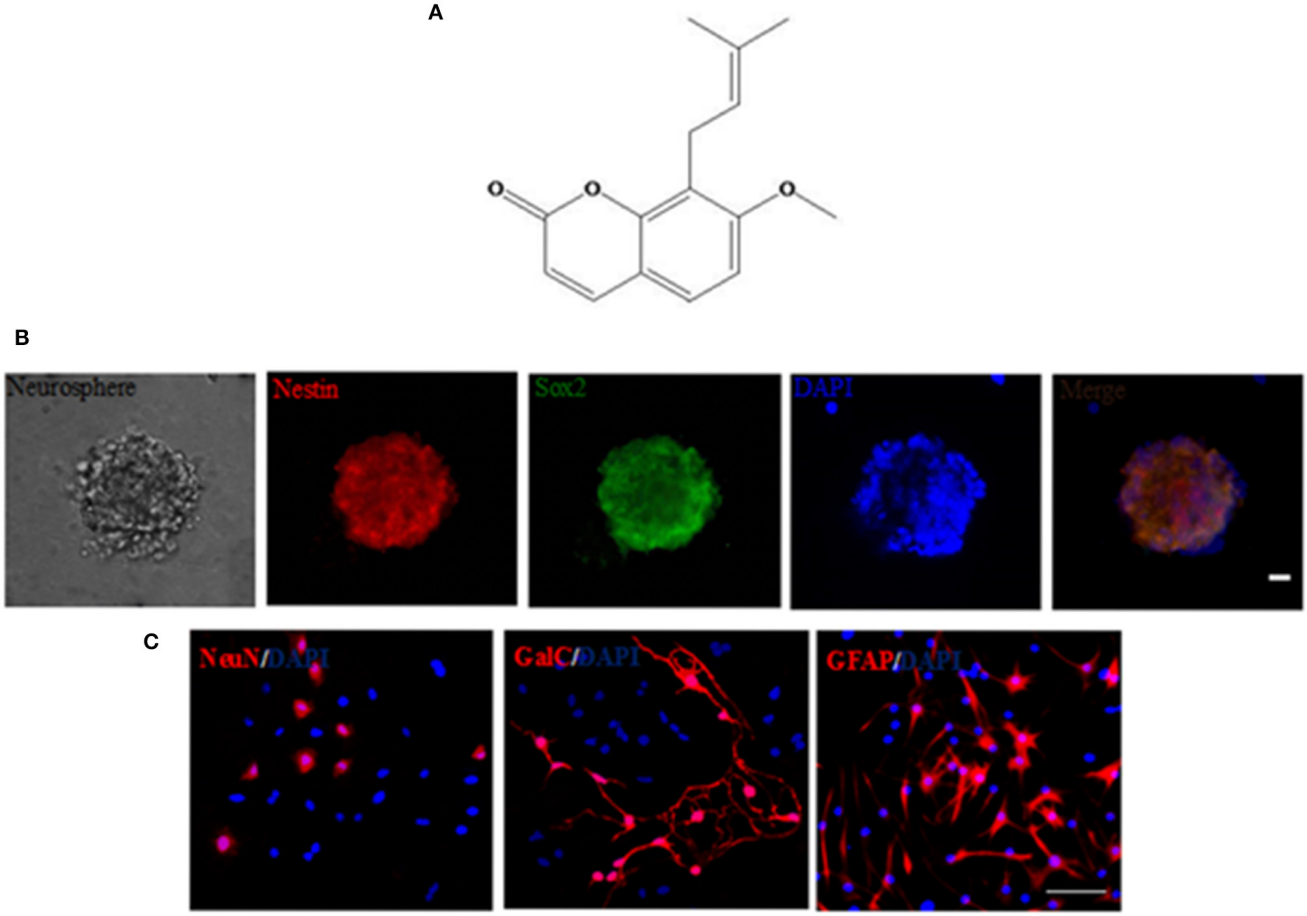
Generation and characterization of NSCs by immunocytochemistry. **(A)** The chemical structure of osthole. **(B)** A typical neurosphere was identified by immunocytochemistry staining of the neural specific markers Nestin (red) and Sox2 (green), and the nuclears were stained with DAPI (blue). Scale bar = 20 μm. **(C)** Differentiation of NSCs *in vitro*. After 10–14 days of culture, NSCs differentiated into NeuN^+^ neurons, GalC^+^ oligodendrocytes and GFAP^+^ astrocytes by immunostaining, and the nuclears were stained with DAPI (blue). Scale bar = 75 μm.

Ost (structure shown in Figure 1A, purity >98%) was purchased from the National Institute for the Control of Pharmaceutical and Biological products (110822-200407; Beijing, China), dissolved in carboxymethyl cellulose sodium (CMC-Na, 0.05%), and subsequently store at 4°C (Chen et al., 2011; Kong et al., 2015).

### Cell viability assay

The cell counting kitCCK-8 (Dojindo Laboratories; Kumamoto, Japan) was used to measure cell viability according to the manufacturer's instructions. Briefly, 10 μL of CCK-8 solution was added to each well containing 100 μL of cell culture supernatant (10^5^ cells) in 96-well plates. The reaction system was incubated at 37°C for 4 h. Absorbance was measured in a microplate reader (MR-96, Mindray, Shenzhen, China) at 450 nm (Chen et al., 2016; Jiao et al., 2016). The cell viability for each group was calculated by contrasting with the GFP group.

### Flow cytometric analysis of cell apoptosis

NSCs were seeded onto 6-well plates (2 × 10^6^ cell) and cultured at 37°C for 48 h. Subsequently, cells were collected and washed twice with PBS, resuspended in 500 μL of binding buffer and mixed with 5 μL of Annexin V-Light 650, and 5 μL of Propidium Iodide was added to the cells, followed by incubation in the dark for 15 min (Zou et al., 2013; Ma et al., 2014). Apoptotic cells were analyzed using a flow cytometer.

### miR-9 inhibitor transfection and cell differentiation into neurons

APP-expressing NSCs were transfected with antisense miR-9 oligonucleotide (GenePharma, Shanghai, China) using Lipofectamine 2000 reagent according to the manufacturer's instructions (Wang et al., 2011). miR-9 inhibitor: 5′-UCAUACAGCUAGAUAACCAAAGA-3′. Subsequently, cells were divided into five groups: GFP (transduced with GFP), APP (transduced with APP), APP+Ost, APP+miR-9 inhibitor, APP+miR-9 inhibitor+Ost. After 72 h of transfection, NSCs were cultured in differentiation medium composed of DMEM/F12 supplemented with 10% FBS plus 1% P/S for 10–14 days (Zhang et al., 2012). The medium was changed every 3 days.

### Immunofluorescence labeling

Cryosections of brain were fixed with 4% paraformaldehyde, penetrated with 1% Triton X-100, and then washed three times with phosphate-buffered saline (PBS). Sections were incubated with 10% donkey serum albumin (Abcam) in PBS for 30 min, after which primary antibodies were added and incubated at 4°C overnight. The following primary antibodies were used: mouse anti-Nestin (1:150, Abcam, Cambridge, MA, USA), rabbit anti-Sox2 (1:150, Abcam), anti-neuronal nuclei (NeuN, 1:150, Abcam), anti-galactocerebroside (GalC, 1:150, Chemicon), anti-glial fibrillary acidic protein (GFAP, 1:150, Abcam), anti-NF-M (1:150, StemCell Technologies, Vancouver, BC, Canada), anti-APP (1:150), anti-Aβ (1:150, Abcam) and anti-synaptophysin (SYP, 1:150, Abcam). After washing three times with PBS, sections were incubated with Cy3-conjugated donkey anti-rabbit immunoglobulin lgG secondary antibodies (1:200, Jackson, West Grove, PA, USA) for 1 h at room temperature. All of these were then supplemented with DAPI nuclear dye (1:100, Sigma), washed third with PBS, and viewed using an inverted fluorescence microscope (Gu et al., 2015). Image J (National Institutes of Health, Bethesda, MD, USA) was used for quantitative analysis.

### Quantitative real-time polymerase chain reaction

Quantitative real-time polymerase chain reaction (qRT-PCR) was performed as previously described (Li et al., 2016). Total RNA from tissue was extracted using TRIzol reagent (Carlsbad, CA, USA) and quantified using a spectrophotometer. RNA (3 μg) was converted to cDNA by reverse transcriptase using a RevertAidFirst Strand cDNA Synthesis Kit (Thermo Scientific, Lafayette, CO, USA). miR-9 and U6 small nucleolar RNA (Guangzhou RiboBio Co., Ltd, Guangzhou, China) were used for normalization and U6snRNA (U6) served as a control. The qRT-PCR primers used in the present study are described in Table 1. PCR was performed using 2 μL of cDNA and TransStart Top Green qPCR SuperMix (TransGen Biotech, Beijing, China) under the following conditions: 94°C for 30 s; 35 cycles of 94°C for 5 s, 60°C for 15 s, and 72°C for 10 s. The results are expressed as Cq-values. Relative changes in gene expression were determined using the ΔΔCt method, and β-actin was used as an internal control (Ma et al., 2014).

**Table 1:** Mouse Primers for qRT-PCR.

**Genes**	**Sense (5′ → 3′)**	**Anti-sense (5′ → 3′)**
APP	GACTGACCACTCGACCAGCAGGTTCTG	CTTGTAAGTTGGATTCTCATATCCG
Notch1	TCGTGTGTCAAGCTGATGAGGA	GTTCGGCAGCTACAGGTCACAA
Hes 1	GCAGACATTCTGGAAATGACTGTGA	GAGTGCGCACCTCGGTGTTTA
β-actin	GGGAAATCGTGCGTGACCT	TCAGGAGGAGCAATGATCCTG

### Western blot analysis

Equal amounts of proteins (50 μg) were loaded on 10% SDS-PAGE and transferred onto polyvinylidenedifluoride membranes. The membranes were blocked for 1 h with a blocking buffer containing 5% BSA in Tris-buffered saline solution and Tween 20 (10 mM Tris-HCl, 150 mM NaCl, 0.05% Tween 20; TBS-T). Membranes were then incubated overnight at 4°C with different primary antibodies diluted in the same blocking buffer. Incubations with HRP-conjugated secondary antibodies (Sigma) were performed for 1 h at room temperature and visualized by quantitative chemiluminescence using ECL Western blotting detection reagents (Millipore). Signal intensity was quantified using Image J. Antibodies used were as follows: anti-APP (1:1,000, Abcam), anti-Aβ (1:1,500, Abcam), anti-NICD (1:1,000, Abcam), and anti-Hes 1 (1:1,000; Santa Cruz Biotechnology). To control for loading, blots were stripped and reprobed with mouse anti β-actin (1:2,000; Santa Cruz Biotechnology; Smrt et al., 2010).

### Ost treatment of APP_swe_/PS1_ΔE9_ mice

APP/PS1 double transgenic (Tg) mice, overexpressing mutated human APP and PS1 (APP_swe_/PS1_ΔE9_), which could effectively simulate the pathological features of AD patients (McClean and Holscher, 2014), were purchased from the Model Animal Resource Information Platform (Nanjing, China). Tg mice were housed under a 12-h light/dark cycle, with food and water freely available. Cognitive impairment first appeared in 7-month-old Tg mice, and Aβ deposits in the hippocampus could be detected in 9-month-old Tg mice (Zhou et al., 2015). In the present study, Tg mice at an age of 9 months were used and randomly divided into two groups (*n* = 8 for each group): an Ost-treated group (Tg+Ost), intragastrically (i.g.) treated with Ost (20 mg/kg, dissolved in 0.05% CMC-Na) daily for 6 weeks (Kong et al., 2015); wild-type (WT) C57BL/6 mice at the same age served as controls, and were treated with 0.05% CMC-Na (i.g.) daily for 6 weeks. All procedures involving animals were reviewed and approved by the Liaoning University of Traditional Chinese Medicine Institutional Animal Care and Use Committee.

### Morris water maze test

After 6 weeks treatment with Ost, Morris water maze (MWM, Chengdu TME Technology Co., Ltd., Chengdu, China) was used to evaluate learning and memory capacity of the mice following methods previously described (Du et al., 2016). The apparatus consisted of a circular pool (120 cm diameter × 60 cm height) with a black inner wall, which was subdivided into four equal quadrants and filled with water (25°C) to the depth of 30 cm. An escape platform (10 cm diameter) was placed in one of the quadrants (the target quadrant) and submerged ~2 cm below the surface of the water. The test contained a platform trial that measured the animal's spatial acquisition ability and a spatial probe test that assessed memory. All the data, including the swim path and the swim time, were measured by a camera and automated analyzing system.

### Slice preparation

After the MWM test, mice were deeply anesthetized with chloral hydrate and perfused with 1% PBS. Subsequently, 4% paraformaldehyde was perfused to fix the brain. The brains were harvested and cryo-protected in PBS containing 30% sucrose until brains sank to the bottom. Subsequently, the brains were equilibrated to an optimum cutting temperature and placed in the freezer (Zeng et al., 2016). The brains were sectioned into 4–10 μm thick sections using a cryostat (CM1900, Leica). The sections located in the hippocampus were mounted on glass slides for staining and visualized on an OLYMPUS SZX9 and BX51 microscope (Tokyo, Japan) equipped with a digital camera.

### Nissl staining

In this study, 4 μm slices for Nissl staining. The sections (randomly chosen) were processed through different baths in the following order (and time): 100% ethanol (30 s), 95% ethanol (30 s), 70% ethanol (30 s), distilled water (30 s, three times), cresyl violet (56°C, 1 h), neutral differentiation solution (2 min), 100% ethanol (30 s), xylene (1 min); the samples were then mounted with neutral balata and covered with a coverslip (Zhai et al., 2015). The Nissl staining sections were visualized on a OLYMPUS SZX9 and BX51 microscope (Tokyo, Japan) with a digital camera.

### Statistical analysis

All data are expressed as the means ± standard deviation (SD) and were analyzed using SPSS version 13.0 (SPSS, IL, USA). Differences of other parameters were evaluated using the analysis of variance for multiple groups. Differences were considered significant at *P* < 0.05.

## Results

### Transduction of GFP and APP into NSCs

To identify the immunocytochemical characteristics of NSCs, derived from the SVZ region of newborn C57BL/6 mice, the neural-specific markers Nestin and Sox2 were utilized. Immunocytochemical labeling showed that cultured NSCs were positive for Nestin and Sox2 (Figure 1B). In addition, NSCs changed morphology and developed into neurons (NeuN^+^), oligodendrocytes (GalC^+^), and astrocytes (GFAP^+^) in NSC differentiation medium after 10–14 days (Figure 1C), indicating that NSCs are multipotent (Oh et al., 2015).

**Figure 2:**
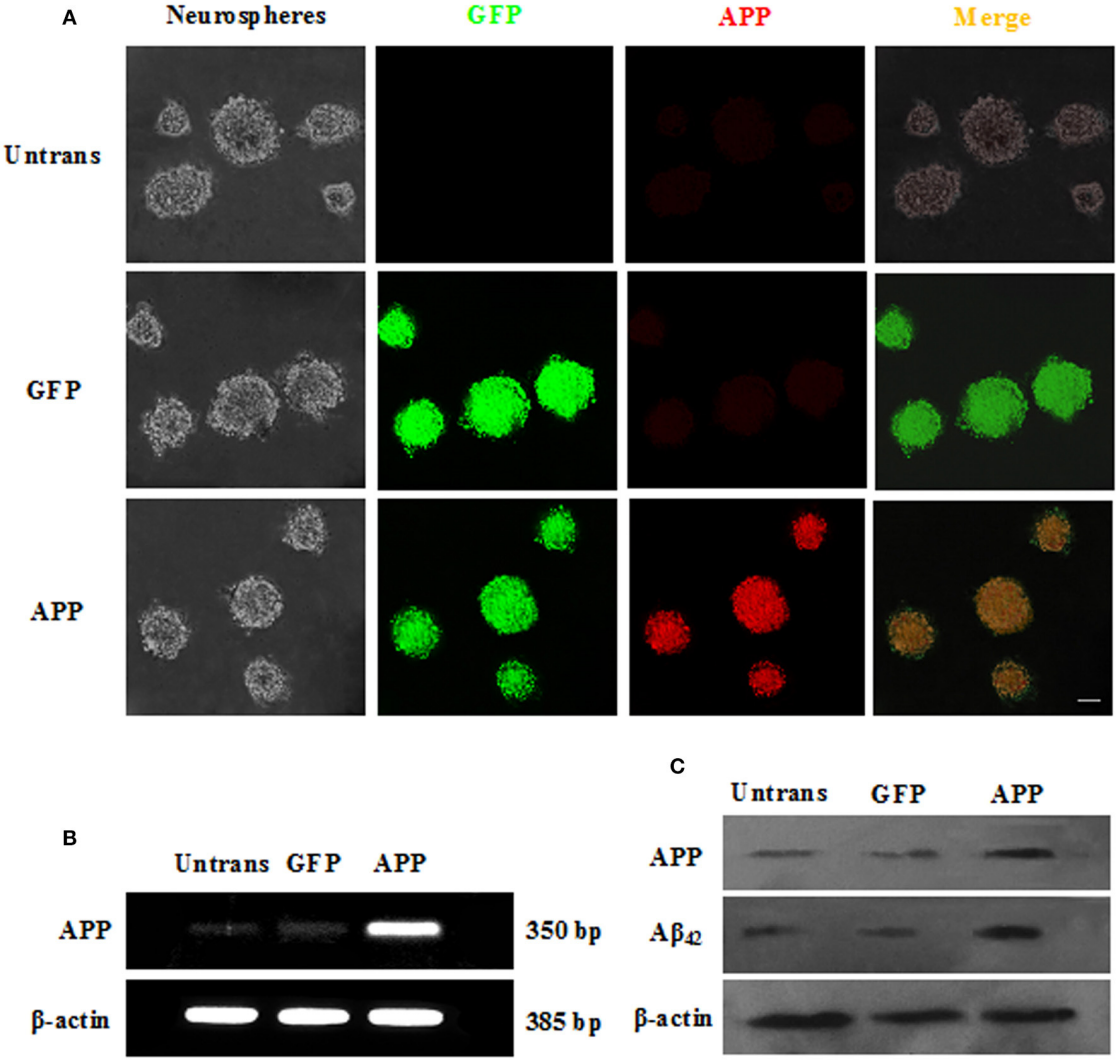
Transduction of NSCs with a single lentiviral vector encoding APP and GFP. **(A)** NSCs transduced with both vectors are GFP^+^ (green), and APP (red) was overexpressed in the APP group but not in the GFP group or untrans groups. Scale bar = 50 μm; **(B)** Expression of APP mRNA was confirmed by RT-PCR; **(C)** APP and Aβ_42_ proteins were measured by Western blotting.

We subsequently transfected these cells with APP/GFP or GFP lentiviral vectors. After 3 days of transduction, strong GFP expression was observed in ~82.3% cells transduced with both vectors, while strong APP staining was visible in the APP group (Figure 2A). RT-PCR and Western blot (Figures 2B,C) revealed the abundant expression of APP, which gives rise to the Aβ, as a neurotoxic oligomer (Haass and Selkoe, 2007; Schonrock et al., 2010b). APP mRNA and protein showed the highest expression in APP-expressing cells than in other groups, but the GFP group showed no difference with the Untrans group.

### Ost promotes cell viability and inhibits cell apoptosis in APP-expressing NSCs

**Figure 3:**
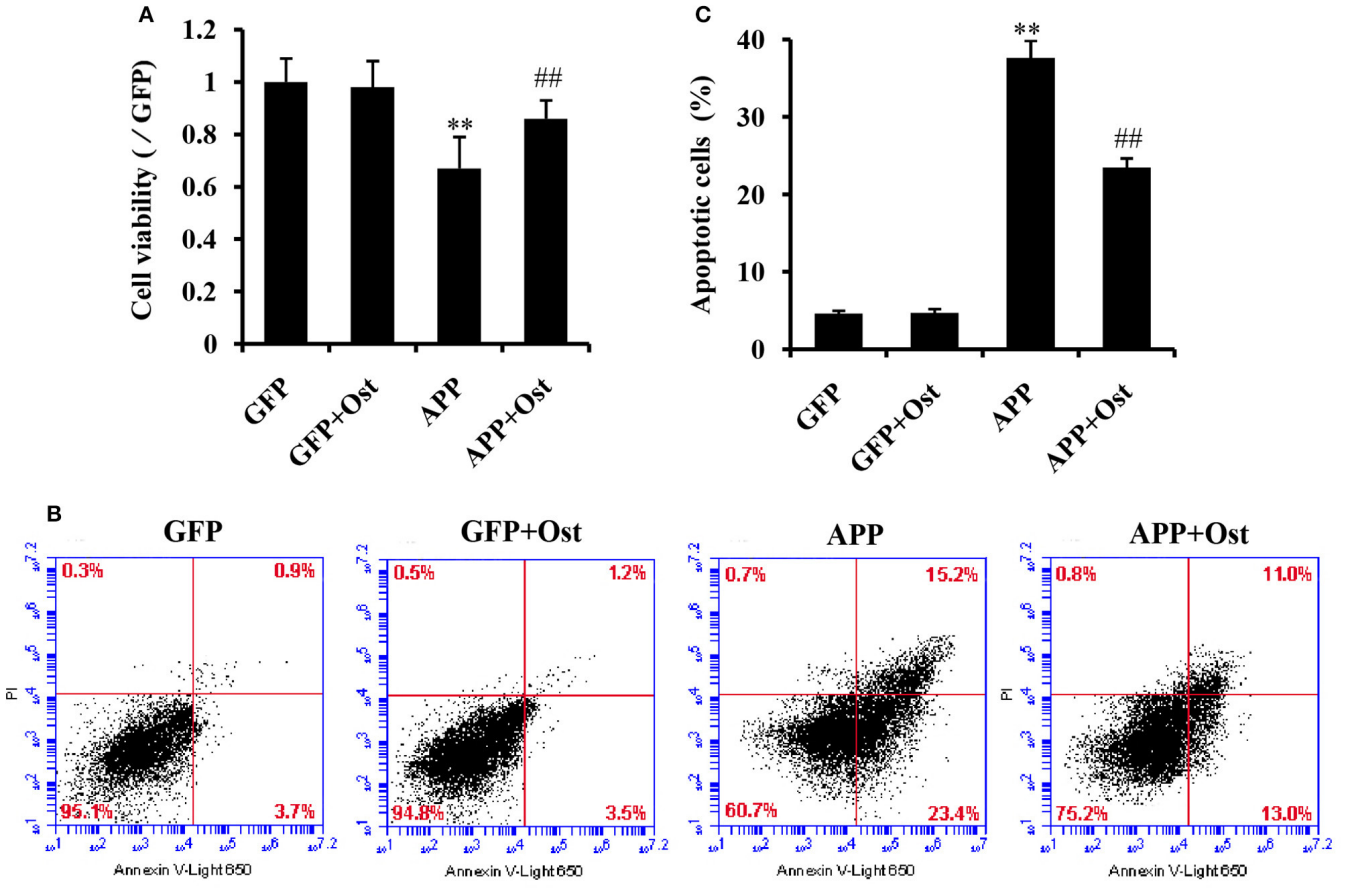
Ost promotes cell viability and inhibits cell apoptosis in APP-expressing NSCs. **(A)** NSCs viability was measured using a CCK-8 assay; **(B)** NSCs apoptosis was detected by flow cytometric analysis; **(C)** Quantitative analysis of apoptotic NSCs. Data represent the means ± SD of three independent experiments. **P < 0.01, comparisons between the GFP group and APP group; ^*##*^*P* < 0.01, comparisons between the APP group and APP+Ost group.

To evaluate whether Ost protects NSCs transduced with APP. APP-expressing NSCs were incubated with Ost (100 μmol/L) for 24 h (Yao et al., 2015), and subsequently, cell viability was determined using a CCK-8 kit. As shown in Figure 3A, APP-expressing NSCs markedly decreased cell viability (67.0 ± 12 vs. 100% in GFP group, *n* = 3, *P* < 0.01), while treatment with Ost obviously restored cell survival to 86.0 ± 6.9% compared to the APP group (*n* = 3, *P* < 0.01, Figure 3A). No significant differences were obtained between the GFP and GFP+Ost groups (*P* > 0.05).

To further investigate the protective effects of Ost on APP-expressing NSCs, cell apoptosis was assayed using flow cytometric analysis. The results showed that cell apoptosis reached 37.6 ± 2.2% in the APP group, a level significantly higher than observed in the GFP cells (vs. 4.6 ± 0.4% in GFP group, *n* = 3, *P* < 0.01, Figures 3B,C), and the addition of Ost resulted in a significant reduction in cell apoptosis (from 37.6 ± 2.21% to 23.5 ± 1.2%, *n* = 3, *P* < 0.01, Figures 3B,C) in the APP+Ost group. However, no change in cell death were observed between the GFP and GFP+Ost groups. Based on the above evidence, we successfully developed an AD cell model and 100 μmol/L Ost had no cytotoxicity (Lee et al., 2013).

### Ost increases the differentiation of APP-expressing NSCs into neurons by upregulating miR-9

**Figure 4:**
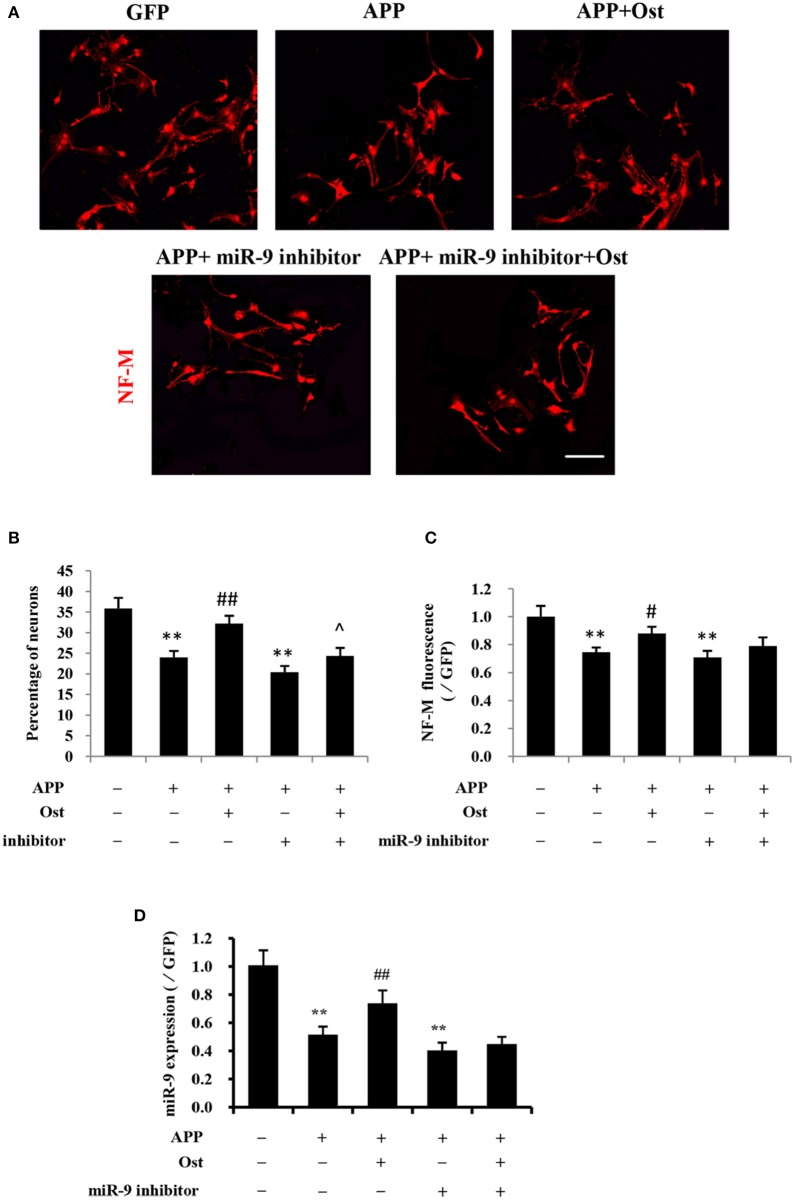
Ost promotes neuronal differentiation via increasing miR-9 in APP-expressing NSCs. **(A)** Neuronal differentiation was indicated by NF-M (red) staining. Scale bar = 100 μm. **(B)** Neuronal differentiation was quantified as the percentage of NF-M-positive cells; **(C)** Quantitification of the NF-M immunofluorescence intensity. **(D)** The miR-9 expression was quantitatively analyzed by real-time PCR. Relative changes in gene expression were determined using the ΔΔCt method. U6 was included as a loading control. Data represent the means ± *SD* of three independent experiments. **P < 0.01 vs. the GFP group; ^*##*^*P* < 0.01 vs. the APP group; ^∧^*P* < 0.05 vs. the APP plus miR-9 inhibitor group.

At 10–14 days after NSCs were cultured under differentiation conditions, cells were fixed and immunostained with antibodies against Neurofilament M (NF-M; Yang et al., 2014; Xia et al., 2015). Here, immunofluorescence analysis revealed that the proportion of NF-M immunoreactive cells was 24.0 ± 1.6% in the APP group, which was lower than that in the GFP group, at 35.9 ± 2.5%, but Ost treatment significantly increased the percentage of NF-M positive cells (32.2 ± 1.9% vs. APP group, *n* = 3, *P* < 0.01, Figures 4A,B). Moreover, the NF-M-positive fluorescence signal was significantly decreased in the APP group compared with that in the GFP group (74.6 ± 3.4% vs. 100%, *n* = 3, *P* < 0.01), while Ost (88.0 ± 4.9%) treatment increased NF-M expression (*n* = 3, *P* < 0.01, Figure 4C), further confirming that Ost stimulated NSCs differentiation into neurons in an AD cell model.

To investigate whether Ost treatment could regulate the expression of miR-9 in APP-expressing cells, a miRNA qRT-PCR assay was used. In the present study, APP transduction led to the inhibition of miR-9 expression in these cells (0.57 ± 0.03 in the APP group vs. 1.00 ± 0.11 in the GFP group, *n* = 3, *P* < 0.01, Figure 4D). By contrast, Ost treatment resulted in the upregulation of miR-9 (0.90 ± 0.03 APP+Ost group vs. 0.57 ± 0.03 APP group, *n* = 3, *P* < 0.01, Figure 4D). To further investigate the role of miR-9 in the present study, we used an antisense miR-9 oligonucleotide to inhibit miR-9 (Wang et al., 2011). As shown in Figure 4D, miR-9 inhibitor significantly reduced the expression of miR-9, and neuronal differentiation was significantly decreased in the APP plus miR-9 inhibitor group (*n* = 3, *P* < 0.01, Figures 4A–C). Treatment with Ost partially increased the percentage of NF-M positive cells in the APP plus miR-9 inhibitor group (24.3 ± 1.98% vs. 20.43 ± 1.50, *n* = 3, *P* < 0.05, Figures 4A,B).

### Ost increases the differentiation of APP-expressing NSCs into neurons by inhibiting the notch signaling pathway

**Figure 5:**
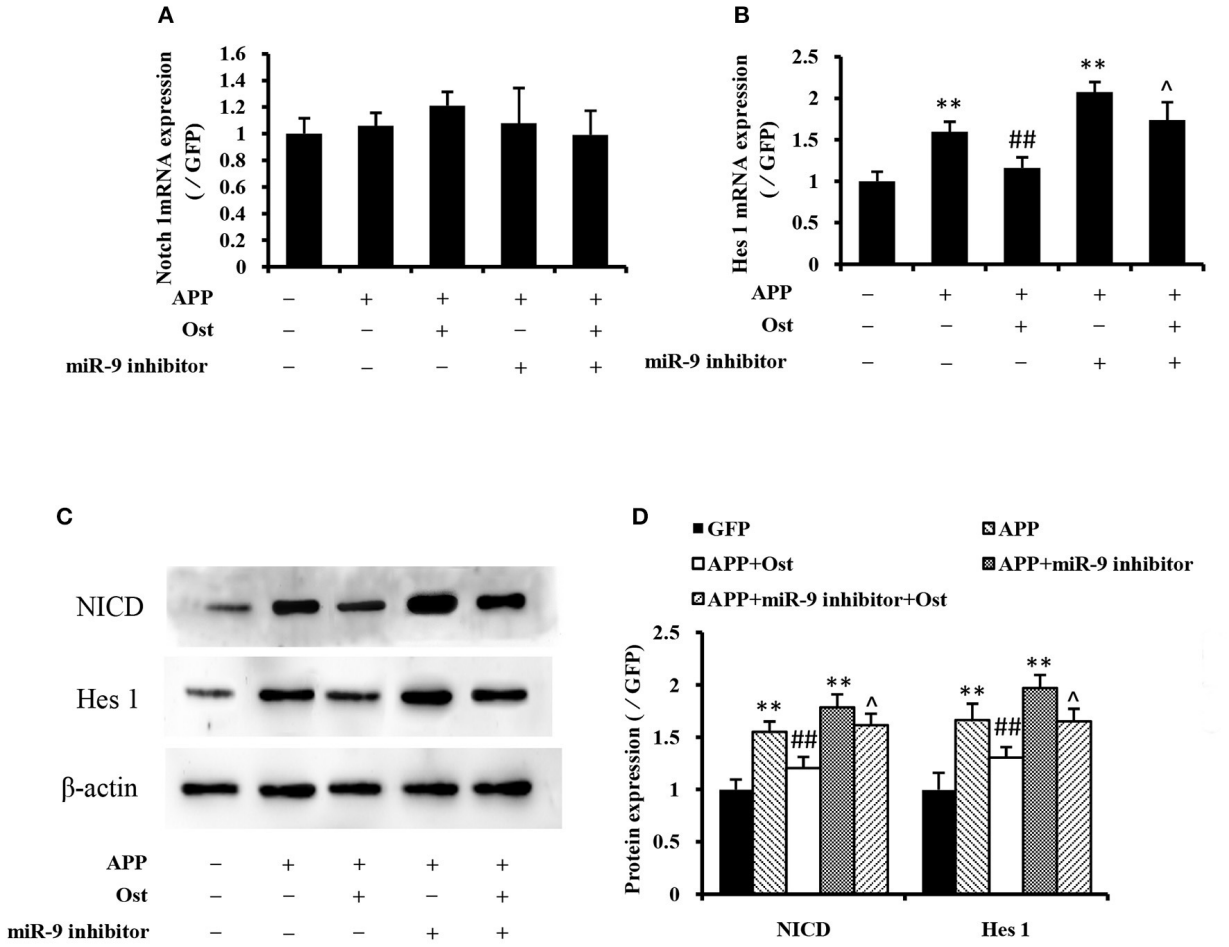
Effects of osthole and miR-9 on the Notch signaling pathway expression. The mRNA levels of Notch 1and Hes 1 were quantitatively analyzed by real-time PCR. Relative changes in gene expression were determined using the ΔΔCt method, and β-actin was used as an internal control **(A,B)**. **(C)** Protein levels of NICD and Hes1 were detected by Western blotting after osthole treatment or miR-9 inhibitor transfection or the combination. **(D)** Quantification of protein expression using Image J software, and normalized using β-actin as an internal control. **P < 0.01 vs. the GFP group; ^*##*^*P* < 0.01 vs. the APP group; ^∧^*P* < 0.05 vs. the APP plus miR-9 inhibitor group.

Several studies reported that APP could increase glial differentiation and decrease neuron differentiation of NPCs associated with the activation of Notch signaling (Kwak et al., 2011). The qRT-PCR results showed that APP-expressing NSCs did not change the expression of Notch 1 while the expression of Hes 1 was drastically increased compared with the GFP group (*n* = 3, *P* < 0.01, Figures 5A,B), indicating that APP-induced Notch signaling activation does not involve up regulation of Notch 1 expression. Treatment with Ost attenuated the increase of Hes 1 expression compared with the APP group (*n* = 3, *P* < 0.01, Figure 5B). Western blot analysis was performed to verify the levels of NICD and Hes 1 protein expression in these cells. These results shown in Figures 5C,D suggested that infection of APP significantly increased the protein levels of NICD and Hes 1 by 155.47 ± 9.69 and 166.68 ± 15.47% (*P* < 0.01), respectively, compared with the GFP group. Meanwhile, treatment with Ost reduced the protein levels of NICD and Hes 1 by 120.81 ± 10.50 and 130.71 ± 9.98% (*n* = 3, *P* < 0.01), respectively.

Inhibition of miR-9 led to increased levels of endogenous Hes 1 mRNA and NICD, Hes 1 proteins compared with the GFP group (*P* < 0.01, Figure 5), while treatment with Ost significantly reduced the levels of endogenous protein compared with the APP plus miR-9 inhibitor group (*n* = 3, *P* < 0.05). These results suggest that Ost stimulates APP-expressing NSCs differentiate to neurons partly through upregulation of miR-9 and inhibition of the Notch signaling pathway in APP-expressing cells.

### Ost improved the learning and memory deficits in APP/PS1 double Tg mice

**Figure 6:**
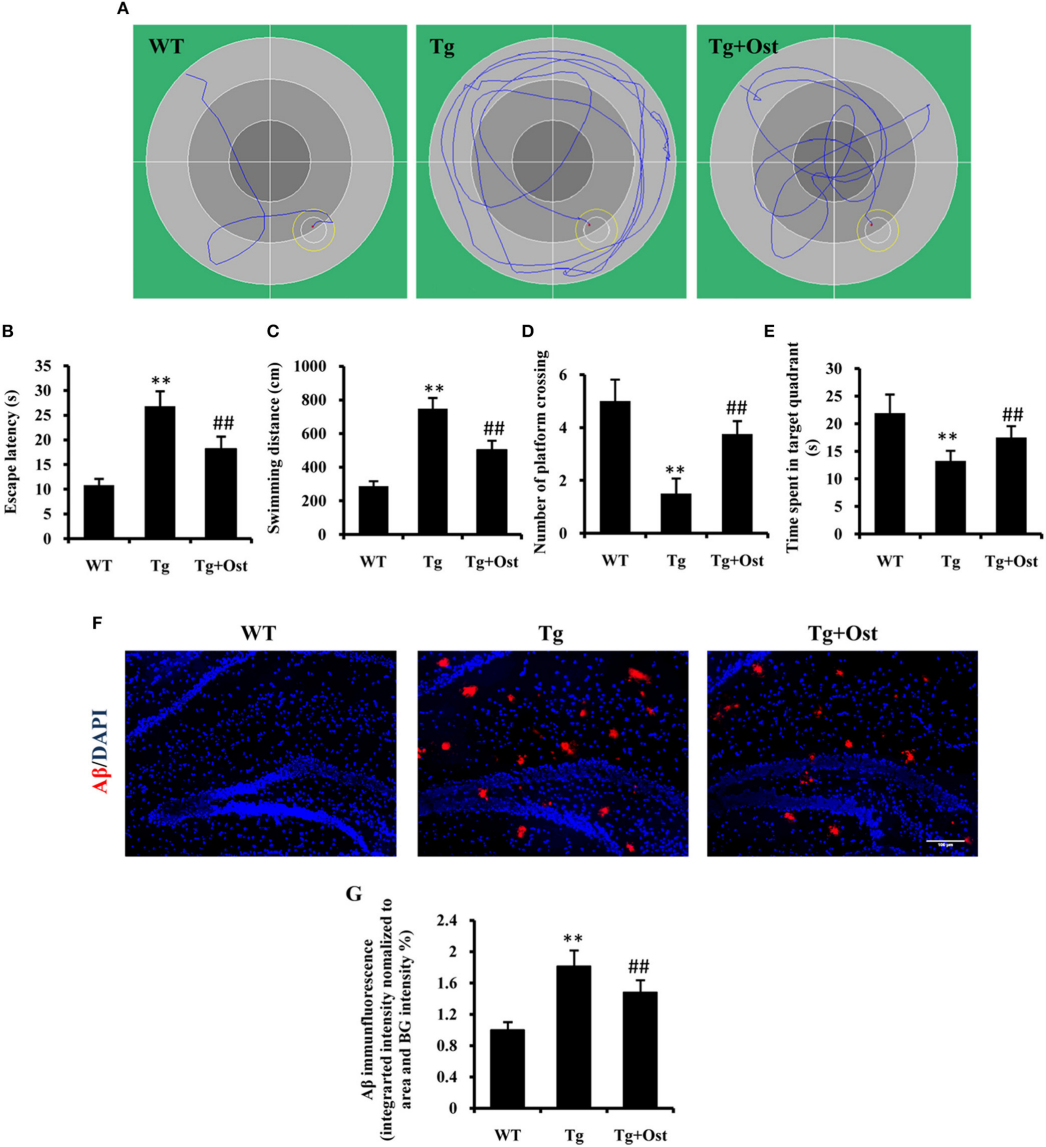
Ost treatment improved the learning and memory deficits in the Morris Water Maze and reduced Aβ accumulation in Tg mice. **(A)** Typical individual swim paths at day 5. **(B)** Escape latency to find the hidden platform from day 1 to 5. **(C)** Swimming distance to find the hidden platform from day 1 to 5. **(D)** The number of platform crossings. **(E)** Time spent in the target quadrant in the probe trial. **(F)** Aβ deposition in the hippocampus and the cortex was analyzed after immunofluorescence staining, scale bar = 100 μm. **(G)** Quantification of the Aβ immunofluorescence intensity. **P < 0.01, comparisons between the WT group and Tg group; ^*##*^*P* < 0.01, comparisons between the Tg group and Tg+Ost group (*n* = 8).

To investigate the potential protective effects of Ost on the cognitive function of Tg mice, we first examined the effect of Ost on the water maze task. Spatial learning was expressed as the latency of distance and time spent on finding the escape platform in the water maze. During the navigation test, the typical swimming paths of the WT group, Tg and Tg + Ost groups were observed (Figure 6A). In the testing period, Tg mice showed that the latency to find the platform and the escape distance were significantly longer than observed for WT mice (*n* = 8, *P* < 0.01, Figures 6B,C). For treated Ost mice, the escape latency and escape distance were significantly decreased compared with Tg mice in the testing period (*n* = 8, *P* < 0.01, Figures 6B,C).

The platform was removed in the probe trial to measure the memory function on the 5th day of the test. The results indicated the number of platform crossings was notably reduced for Tg mice compared to WT mice, and the time spent in the target quadrant was significantly shorter for Tg mice than for WT mice (*n* = 8, *P* < 0.01 vs. the WT group, Figures 6D,E). In the Tg+Ost group, the number of platform crossings and time spent in the target quadrant were significantly increased compared to the Tg group (*n* = 8, *P* < 0.01 vs. the Tg group, Figures 6D,E). These results suggested that Ost treatment improved the learning and memory ability of APP/PS1 Tg mice.

### Ost reduced Aβ accumulation in APP/PS1 double Tg mice

Several studies have reported that Aβ accumulation is the major causative factor in AD development (Schonrock et al., 2010a). Therefore, we examined Aβ accumulation in APP/PS1 Tg mice. We confirmed a higher accumulation of Aβ in the hippocampus of APP/PS1 Tg mice using immunofluorescence (*n* = 8, *P* < 0.01 vs. WT group, Figures 6F,G), and Ost treatment reduced Aβ accumulation in the hippocampus compared to Tg mice (*n* = 8, *P* < 0.01 vs. the Tg group, Figures 6F,G).

### Ost promoted neuronal survival and increased the synaptic proteins levels in APP/PS1 double Tg mice

**Figure 7:**
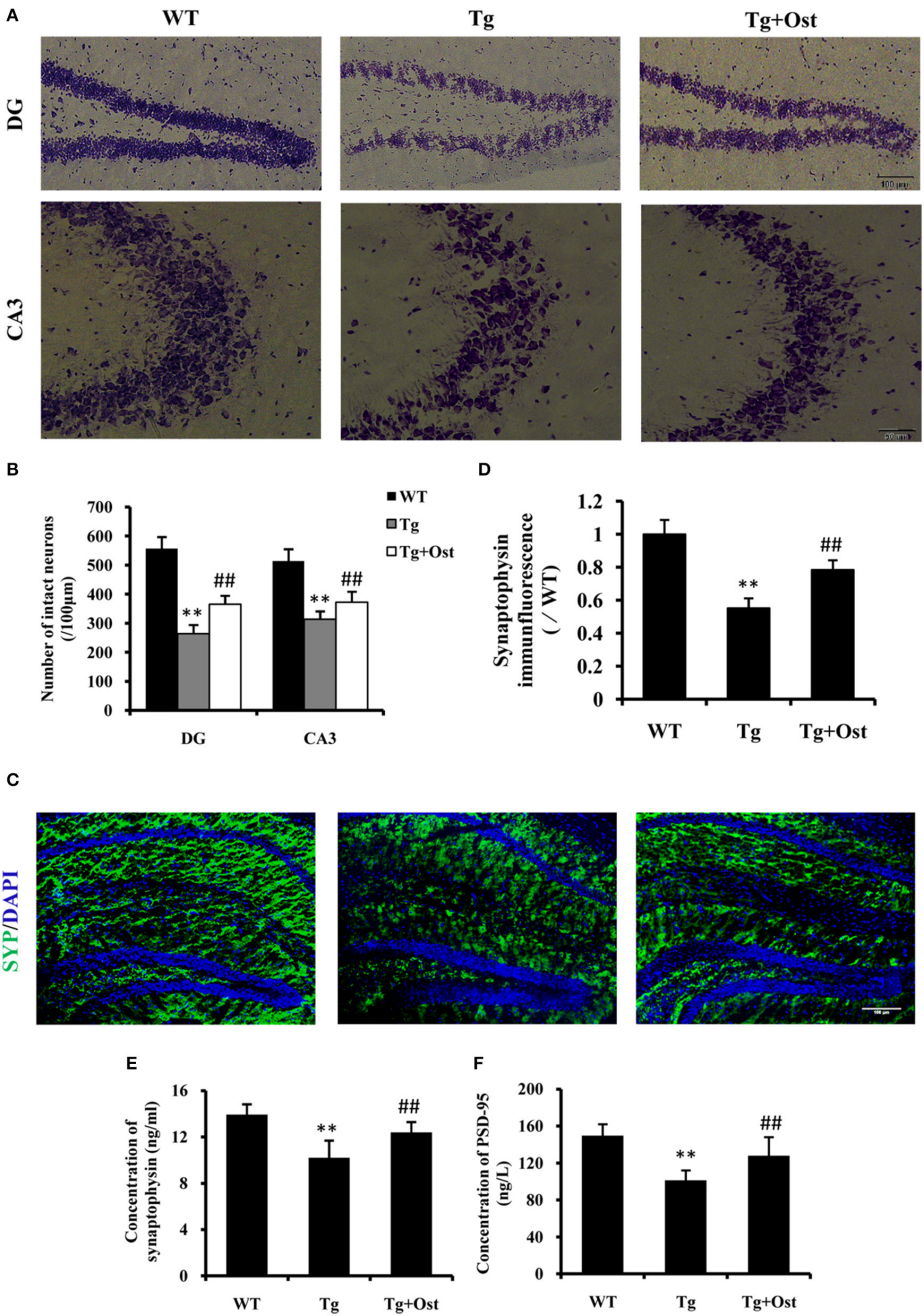
Ost promoted neuronal survival and increased the synaptic protein levels in APP/PS1 Tg mice. **(A)** Representative photographs of Nissl-stained hippocampal DG and CA3 from brain sections (scale bar = 100 μm); **(B)** Number of neurons in the hippocampal area (DG and CA3). **(C)** Immunocytochemistry for synapyophysin (red) and DAPI (blue) in the hippocampus. Scale bar = 100 μm. **(D)** Quantification of the SYP immunofluorescence intensity. SYP **(E)** and PSD-95 **(F)** concentrations measured using ELISA assay. **P < 0.01 vs. the WT group; ^*##*^*P* < 0.01 vs. the Tg group (*n* = 8).

The loss of neurons in the hippocampus area is one of characteristics of AD (Haass and Selkoe, 2007). Nissl staining revealed that the number of neurons was significantly reduced in the hippocampal area, including DG and CA3, compared to the WT group (264.0 ± 30.3 vs. 557.0 ± 40.1 cells in the DG; 313.8 ± 27.4 vs. 513.8 ± 41.4 cells in the CA3, *P* < 0.01, *n* = 8, Figures 7A,B). Treatment with Ost increased the number of neuronal cells in the hippocampal area (DG: 365.0 ± 30.2 cells; CA3: 372 ± 37.2 cells, vs. the Tg group, *P* < 0.01, *n* = 8, Figures 7A,B).

To further investigate the effect of Ost on neurons in Tg mice, immunofluorescence was used to analyze the expression of SYP (SYP) firstly. In the present study, the Tg group exhibited a significant reduction in the intensity of SYP in the hippocampus (55.3 ± 5.8%, *v*0.01) compared to the WT group (100%, *n* = 8, Figures 7C,D), while treatment with Ost attenuated the reduction of SYP and obviously restored the protein (78.4 ± 5.8%, *P* < 0.01 vs. Tg group, *n* = 8, Figures 7C,D). Subsequently, we also measured the concentration of SYP and PSD-95 using ELISA. The results showed that Ost increased the levels of SYP and PSD-95 compared to the Tg group (SYP: 12.40 ± 0.89 vs. 10.20 ± 1.49 ng/mL; PSD-95: 127.87 ± 20.20 vs. 101.31 ± 10.72 ng/L, *P* < 0.01, *n* = 8, Figures 7E,F). These data showed that Ost rescues the impairment of hippocampal neurons in APP/PS1 transgenic mice.

### Ost upregulated miR-9 in APP/PS1 double Tg mice

**Figure 8:**
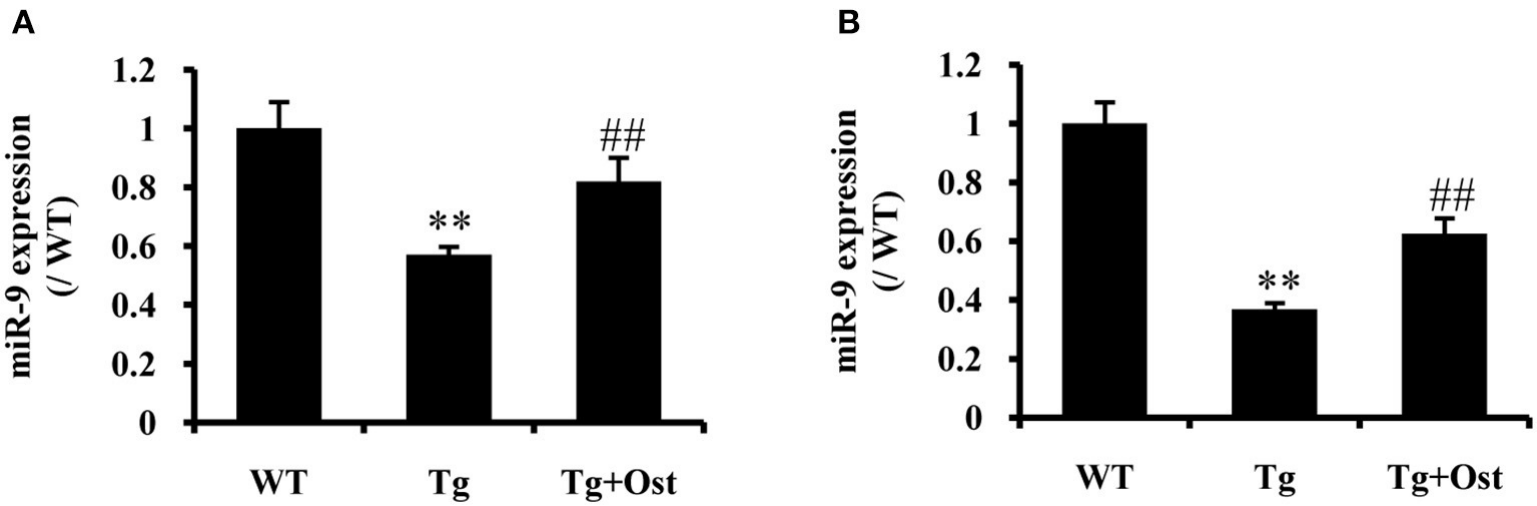
Ost treatment increased miR-9 expression in APP/PS1 Tg mice. **(A)** miR-9 expression in the DG regions. **(B)** The miR-9 expression in the CA3 regions. Data represent the means ± SD of three independent experiments. **P < 0.01 vs. the WT group; ^*##*^*P* < 0.01 vs. the Tg group (*n* = 8).

To investigate the neuroprotective mechanism of Ost in APP/PS1 transgenic mice, qRT-PCR was used to examine miR-9 expression. The results indicated that Ost significantly upregulated miR-9 expression in the DG and CA3 regions (*n* = 8, *P* < 0.01, Figure 8), which was consistent with the *in vitro* result.

## Discussion

In recent decades, the pathogenic mechanisms of AD have been obscure, but the most recognized mechanism is that APP is initially cleaved by β-secretase and subsequently cleaved by γ-secretase generating Aβ oligomers (Mangialasche et al., 2010; Schonrock et al., 2012), which strongly induce neuronal toxicity, synaptic failure, and memory loss in cell or animal models of AD (Selkoe, 2002; Haass and Selkoe, 2007). Recently, numerous studies have revealed the crucial role of APP in AD in mouse neuronal stem cells, neurons, and human neuroblastoma cell lines SH-SY5Y and La-N-1 (LeBlanc et al., 1992; Li et al., 2016). Herein, we also established a cell model of AD by expressing APP by transfecting human APP_695swe_ into mouse NSCs as previously described (Yao et al., 2015).

A growing body of reports have indicated that Ost exerts neuroprotective effects on the development and progression of neurodegenerative diseases. For example, pretreatment with Ost has a protective effect on reducing the loss of cells and inhibiting ROS production in PC12 cells induced using 1-methyl-4-phenylpyridinium ion (MPP^+^) as a neurotoxin to mimic Parkinson's disease (Liu et al., 2010). Consistently, Hu et al. also showed that Ost possesses the ability to prevent exogenous Aβ-induced neuronal cell death by enhancing CREB phosphorylation (Hu et al., 2013). Similarly, previous studies have revealed the neuroprotective effects of Ost on treating traumatic brain injury by reducing neuronal apoptosis, cerebral edema and improving neurological function as a result of its anti-inflammatory and anti-apoptotic abilities (He et al., 2011; Xia et al., 2015). The results of the current study provide evidence that Ost enhanced cell viability and prevented cell death in APP-expressing NSCs.

miR-9 is one of the most enriched miRNAs in the central nervous system of mammals (Landgraf et al., 2007) and induces neurogenesis in varied models (Krichevsky et al., 2006; Zhao et al., 2009; Yang et al., 2013). An increasing number of reports have indicated that miR-9 promotes neural differentiation of MSCs and NSCs by targeting Hes 1, STAT3, Zfp521, FoxG, or 1BAF53a (Krichevsky et al., 2006; Shibata et al., 2008; Yoo et al., 2009; Bonev et al., 2012; Rui et al., 2012). However, notably, miR-9 expression was reduced in both neurons after Aβ treatment and in the brains of AD patients (Schonrock et al., 2010a), and a host of observations showed that miR-9 could be used as a strategy to improve AD. Chang et al. showed miR-9 overexpression attenuates Aβ_42_-induced synaptotoxicity by targeting CAMKK2 (Chang et al., 2014). Moreover, the existence of miR-9 can retard the generation of Tau in the early stages of AD by inhibiting SIRT1 (Charlotte et al., 2012). Consistent with this opinion, the results demonstrated that Ost significantly increased miR-9 expression in APP-expressing NSCs (Figures 3A–C), suggesting that Ost may promote APP-expressing NSCs differentiation into neurons via the upregulation of miR-9.

The Notch signaling pathway is involved in nervous system development and the regulation of stem cells biological activities. Notch signaling pathways were activated through two neighboring cells interactions, accompanied by cleavage to release the Notch intracellular domain (NICD). Subsequently, NICD translocates to the nucleus and regulates the transcription of target genes, such as Hes 1, through its association with CSL proteins (Lathia et al., 2009). Some interesting articles have indicated that miR-9 promotes the differentiation of stem cells into neurons by the Notch signaling pathway, as Notch 1 and hes1 are the target genes of miR-9 (Jing et al., 2011; Tan et al., 2012). Another interesting study displayed that APP increased glial and decreased neuronal differentiation of neural progenitor cells through activation of the Notch signaling pathway (Kwak et al., 2011). Thus, we proposed that there is a cross-talk between miR-9 and the Notch signaling pathway in AD models. In the present study, increased NICD generation and Hes 1 expression associated with fewer neurons in APP-expressing NSCs were observed, indicating that APP transduction reduced the number of neurons through activation of the Notch signaling pathway. However, Ost may promote NSCs differentiation into neurons via upregulation of miR-9 and subsequent inhibition of the Notch signaling pathway in APP-expressing cells.

To further investigate the effect of Ost on neurogenesis by upregulation of miR-9 and subsequent inhibition of the Notch signaling pathway, an antisense miR-9 oligonucleotide was used to inhibit expression and function of miR-9. The results indicated the neuronal differentiation rate of miR-9 inhibitor transfection in the APP group was much lower than that in the GFP and APP groups, accompanied by an increase in NICD and Hes 1 expression, while Ost partially reversed the reduction of neuronal differentiation, accompanied by a reduction of NICD and Hes 1 expression. These observations confirmed that the promotion of neuronal differentiation by Ost is attributed to an increase in miR-9 expression.

To fully confirmed the effect of Ost on neuroprotection in the brain, we used APP/PS1 Tg mice, which can effectively simulate the pathological features of AD patients (McClean and Holscher, 2014). Herein, we demonstrated a preventive effect after 6 weeks of Ost treatment from the age of 6–7 months in APP/PS1 mice, improving cognition and reducing many AD-associated biomarkers. The results indicated that Ost can significantly ameliorate the learning and memory deficits of mice by reducing the escape latency and the distance to goal, increasing the number of platform crossings and prolonging the time spent in the target quadrant (Figure 6) in the MWM test, consistent with previous reports (Liu et al., 2015). We also examined some biomarkers of AD in the hippocampus of APP/PS1 Tg mice, which is a neurogenesis area in the mammalian brain (Braak et al., 1993). Aβ plaques were observed by immunostaining in the hippocampus, and the inhibition of Aβ production by Ost was also examined. Second, to detect whether Ost can rescue the impairment of hippocampal neurons in APP/PS1Tg mice, Nissl staining, immunohistochemistry and ELISA were used. The results showed that the number of DG and CA3 neurons as well as the concentration of synaptic proteins in the Tg group sharply decreased, while Ost treatment increased the number of neurons and the concentration of synaptic proteins in Tg mice, partly through upregulation of miR-9.

In conclusion, this collective evidence clearly demonstrated that Ost promotes cell survival and reduces cell apoptosis in NSCs by APP transduction. Ost also increases the differentiation of APP-expressing NSCs into neurons through upregulation of miR-9 and inhibition of the Notch signaling pathway. *In vivo* study, we also observed that Ost improves cognitive function, decreases the Aβ formation, promotes neuronal survival and synaptic proteins levels. Thus, the findings presented in the present study provided novel insights into the neuroprotective actions and neurobiological mechanism of Ost against AD, suggesting that Ost may be a potential agent for halting disease progression of AD.

## Availability of data and materials

Data supporting the conclusions of this article are presented within the manuscript.

## Author contributions

JY and SL designed and performed the research experiments, drafted, revised, and completed the final version of the manuscript. YY, HL, YX, PG, LW, JY, and SL helped carry out experiments in addition to analyzing and interpreting gathered data. All authors have read and approved of the final version submitted for publication.

### Conflict of interest statement

The authors declare that the research was conducted in the absence of any commercial or financial relationships that could be construed as a potential conflict of interest. The reviewer GHD and handling Editor declared their shared affiliation, and the handling Editor states that the process met the standards of a fair and objective review.
